# Uncommon Denominators

**DOI:** 10.3201/eid1312.000000

**Published:** 2007-12

**Authors:** Polyxeni Potter

**Affiliations:** * Centers for Disease Control and Prevention, Atlanta, Georgia, USA

**Keywords:** Zoonotic diseases, census, art and science connection, humanities and science, Roman art, about the cover

**Figure:**
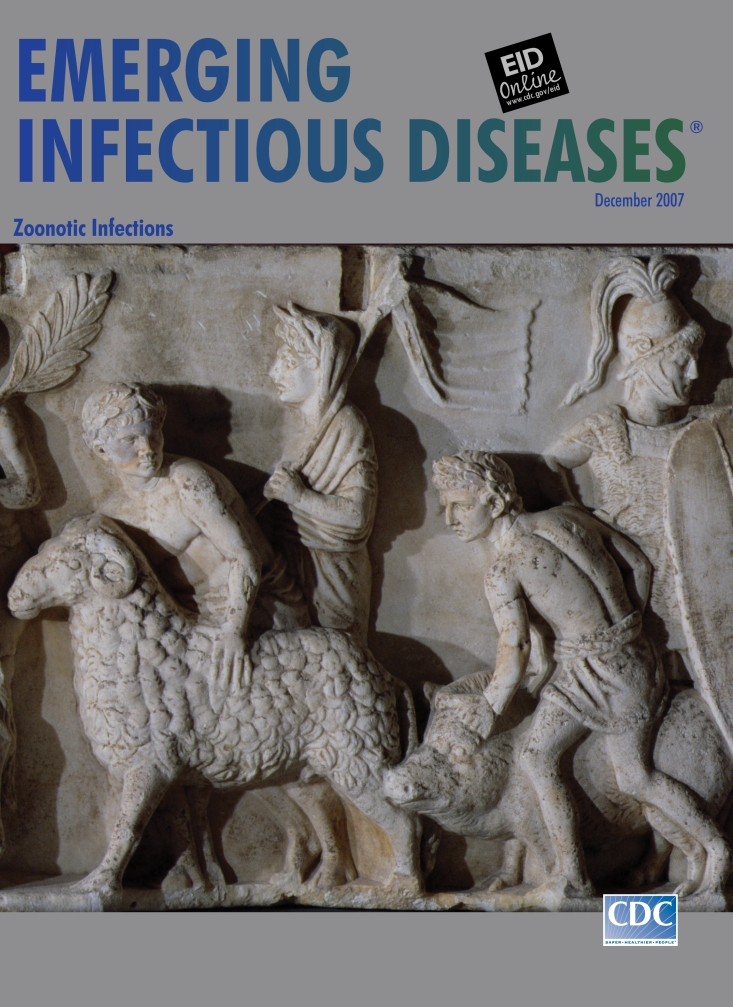
**Census Proceedings on the Campus Martius. Altar of Domitius Ahenobarbus. Decoration from the base of a statuary group. Rome. End of second century BCE.** Marble (78 cm × 559 cm). Louvre, Paris, France/Lauros/Giraudon/The Bridgeman Art Library Nationality/copyright status: out of copyright

“Others will have greater skill for getting the breath of life to spring from bronze more fluidly .... But as for you, Roman, remember to impose your power upon nations. Your art is to decree the rules of peace, to spare the vanquished and subdue the vainglorious,” advised Virgil in the Aeneid, placing himself in the service of imperial ideology (*1*). This was the reign of Augustus (27 bce–14 ce), the first and among the most influential of Roman emperors, who enlisted literature and the arts in support of the new order.

Art of the Roman Empire, from Romulus to Constantine the Great, a period of more than 1,000 years, was expansive and diverse like the Empire itself but left few records of artists or patrons (*2*). Influenced by the Etruscans, the preceding dominant culture in Italy, and the Hellenistic world through colonies in southern Italy and Sicily, its growth awaited the evolution of political institutions during the latest period of Republican history (*3*). Before the conquest of Syracuse, wrote Plutarch, a leading thinker of the Empire’s golden age, “Rome neither had nor even knew” of these refined things, “nor was there in the city any love of what was charming and elegant; rather it was full of barbaric weapons and bloody spoils” (*4*).

Hellenistic influences continued as artists were brought to Rome to repair crumbling monuments and design new ones. Hellenic bronze statues were widely copied, usually in marble. Classicism gave way to a more realistic style, particularly in portrait busts, which were very popular. Art became secular and utilitarian. Architecture flourished on a grand scale, and the vault and dome were invented. Augustus is said to have boasted that he “found Rome of brick and left it of marble” (*5*). The discovery of concrete made possible such monumental buildings as the Pantheon in Rome, which still stands. The triumphant arch, also an invention of the period, exemplified Roman civic and commemorative architecture.

In the Augustan era, sculpture still showed the idealism of Hellenic models, even relief sculpture: shallow three-dimensional carvings on arches, friezes, altars, and other flat areas of temples and public buildings. But the content of reliefs favored the historical and commemorative, intending to narrate in detail triumphant military campaigns and promote the goals of the Empire. In his Ars Poetica, Horace supported this philosophy, as he argued the superiority of painting over any other form of communication to affect and manipulate: “Less vividly is the mind stirred by what finds entrance through the ears than by what is brought before the trusty eyes, and what the spectator can see for himself” (*6*). Public art of the Empire aimed to “write conquerors and conquered in one community” (*7*).

The remains of an altar believed to have been set up in the Campus Martius by Domitius Ahenobarbus, father of Emperor Nero, provide a glimpse into civic commemorative art of the Empire. The Campus Martius (Field of Mars) was a public area of Rome used for military activities; as such, it was dedicated to Mars, god of war and father of Romulus and Remus, legendary founders of the city. The month March (Martius) was named after him, and the Romans called themselves “sons of Mars.” The Campus later became the site of triumphant parades and celebrations and was filled with temples and public buildings.

Contiguous panels of the relief on this month’s cover have the feel of narrative stream. During the census proceedings, a collection of citizens, among them military men serving as guards, are taking part in a religious rite, the *suovetaurilia* (from *sus* [pig], *ovis* [ram], *taurus* [bull]): a ceremony during which livestock were sacrificed to the gods. “Father Mars, I pray and beseech thee that thou be gracious and merciful to me, my house, and my household,” read the Latin prayer (*8*). The sacrifice, whose purpose was purification, was performed at state ceremonies; during agricultural festivals to drive out evil from the fields and purify new crops; as atonement for ritual errors; before military campaigns; and at the conclusion of the census.

The census was the first and principal duty of the Roman censors, high magistrates in charge of this 5-yearly activity. To carry out the census and the purifications that concluded it, they had the power of summoning the people to the Campus Martius, each tribe separately, by public crier. Each paterfamilias appeared in person to account for himself, his family, and his property upon oath, “declared from the heart” (*9*). A person voluntarily absent from the census was considered *incensus* and risked imprisonment and death.

“It is so hard to find out the truth of anything by looking at the record of the past,” wrote Plutarch; “The process of time obscures the truth of former times, and even contemporaneous writers disguise and twist the truth out of malice or flattery” (*4*). Even art can be used for promotion and persuasion. Yet this census-taking relief, a glimpse of Roman life, did more than serve the purposes of the state. It witnessed one of the foundation stones of Roman civilization; a ritual special to the Romans for it symbolized their status as a *populus*, a people, capable of collective action (*10*).

“We are all, so far as we inherit the civilizations of Europe, still citizens of the Roman Empire,” wrote T.S. Eliot, poet and critic of modern European culture. And while his words may not have universal application, they do call attention to Roman legacy in some of our practices. Certainly we relate to the census. In ancient Rome, the practice served to count citizens and assess military strength and tax revenue. In public health, it helps calculate population density. The number of humans, animals, plants, wildlife, and vectors per unit area influences the spread of communicable diseases and their impact, a tax of its own. And “census numbers” of domestic and wild animals, the denominators used to calculate attack, birth, and death rates, can be strong predictors of zoonotic disease. Once again in the words of T.S. Eliot, “... withered stumps of time ... told upon the walls,” uncover uncommon denominators.
